# Cerebral Hemodynamics and Cognitive Function in Cirrhotic Patients with Hepatic Encephalopathy

**DOI:** 10.1155/2016/8485032

**Published:** 2016-12-21

**Authors:** Yuqing Zhou, Qian Dong, Rong Zhang, Shunfeng Zhou, Linqiang Li, Keran Cheng, Rui Kong, Qiang Yu, Shizan Xu, Jingjing Li, Sainan Li, Jiao Feng, Liwei Wu, Tong Liu, Xiya Lu, Kan Chen, Yujing Xia, Jie Lu, Yingqun Zhou, Chuanyong Guo

**Affiliations:** ^1^Department of Gastroenterology, Shanghai Tenth People's Hospital, School of Medicine, Tongji University, Shanghai 200072, China; ^2^The School of Medicine of Soochow University, Suzhou 215006, China; ^3^The Shanghai Tenth Hospital, School of Clinical Medicine of Nanjing Medical University, Shanghai 200072, China

## Abstract

*Aims.* To investigate cerebral hemodynamics in cirrhotic patients with HE and to observe effects of treatment in cerebral hemodynamics and correlations among ammonia, cerebral hemodynamics, and cognitive function.* Methods*. There were four groups: healthy controls (group 1), cirrhosis without HE (group 2), cirrhosis with MHE (group 3), and cirrhosis with OHE (group 4). Ammonia and cerebral hemodynamics (by TCD) were assessed. Patients in group 3 were subsequently randomized to two subgroups: the control (group A) and the treated (group B, treated with lactulose for two months), and they were retested for ammonia and TCD after treatment. Results. Ammonia, *V*_*m*_, *V*_*d*_, PI, and RI were statistically different before treatment, and ammonia, PI, and RI levels paralleled the severity of HE (*P* < 0.05). In group B, *V*_*d*_ increased and ammonia, PI, and RI declined following treatment (*P* < 0.05), while there were no differences in group A (*P* > 0.05). Correlations were found between ammonia and *V*_*d*_, PI, RI, NCT-A, and DST and also found between *V*_*d*_, PI, RI, and NCT-A and DST (*P* < 0.05).* Conclusions*. This study revealed that cerebral hemodynamics were related to the severity of HE and cerebral autoregulation was impaired. There were tight correlations among ammonia, cerebral hemodynamics, and cognitive function, and, following treatment, cerebral hemodynamics improved.

## 1. Introduction

Hepatic encephalopathy (HE) refers to a clinical syndrome associated with a wide spectrum of neurological or psychiatric abnormalities based on metabolic disorders, which is caused by serious acute/chronic liver dysfunction and/or portal-systemic shunt [1]. HE is associated with a poor prognosis, with a 1-year survival rate of less than 50%, which decreases to less than 25% 3 years following diagnosis [2]. HE includes the subtypes minimal hepatic encephalopathy (MHE) and overt hepatic encephalopathy (OHE). It has been reported that the incidence of MHE in patients with chronic liver diseases is up to 50% [3], with more than 50% of patients diagnosed with MHE reported to develop OHE within 30 months [4]. Patients with MHE do not present with obvious clinical symptoms, demonstrating subtle abnormalities in intelligence, neurological function, and cerebral blood flow by fine intelligence tests or electroneurophysiological examinations [5], and patients with OHE do present with obvious clinical symptoms such as changes in temperament, sleepiness, and even coma. The occurrence of HE increases the length of hospital stay, readmission rates, and risk of death and is associated with substantial personal and economic burden on their family and society [6].

Transcranial Doppler (TCD) is a simple, reproducible, and noninvasive method used to detect continuous, long-term hemodynamics and blood physiological parameters in the major intracranial artery including the middle cerebral artery (MCA) at the base of the skull. Cerebral blood flow and metabolism are closely related to cerebral function and indirectly affect cognitive function [7]. In the present study, TCD was used to observe the changes of cerebral hemodynamics in healthy people and cirrhotic patients. Concurrently, we assessed the improvements of cerebral hemodynamics in patients with MHE after two-month oral treatment by lactulose, in order to provide an important basis for the clinical development, diagnosis, and treatment of HE.

## 2. Materials and Methods

### 2.1. Patient Demographics

From August 2015 to April 2016, 92 inpatients and outpatients with cirrhosis of Shanghai Tenth People's Hospital were enrolled in the study. They were aged between 37 and 76 years and included 54 males and 38 females with between 3 and 16 years of education. In etiology, there were 40 cases with B viral hepatitis cirrhosis, 3 cases with C viral hepatitis cirrhosis, 7 cases with primary biliary cirrhosis, 8 cases with autoimmune hepatitis cirrhosis, 8 cases with schistosomiasis cirrhosis, and 26 cases with cryptogenic cirrhosis. Simultaneously, we determined their grades (24 cases of grade A, 36 cases of grade B, and 32 cases of grade C) and scores of Child-Pugh [8] (Table 1). The diagnosis of cirrhosis was made by experienced clinicians and based on clinical signs and symptoms, laboratory tests, and imaging such as ultrasound, CT, and MRI. The diagnosis was consistent with “The Guideline of Prevention and Treatment for Chronic Hepatitis B (2010 version)” [9]. Additionally, 40 healthy volunteers served as controls and were matched with cirrhosis patients according to age (between 35 and 71 years of age), sex (24 males and 16 females), and educational level (between 3 and 16 years of education). The controls had no history of liver diseases, exhibiting normal liver function and morphology. Written informed consent was obtained from all subjects.

Patients with the following conditions were excluded from the study: diseases of the central nervous system (CNS) such as cerebral hemorrhage, cerebral infarction, or mental illnesses. Similarly, patients with alcoholic cirrhosis or drinking history in the past three months were excluded as long-term drinking can cause alcoholic cirrhosis and can also lead to alcoholism encephalopathy, both of which may cause abnormity in TCD. A clinical history of fever in the past one week; fluid, electrolyte, and acid-base disequilibrium in the past two weeks; gastrointestinal bleeding and hemoglobin less than 90 g/L in the past two weeks; taking lactulose, probiotics, and rifaximin in the past two months; taking neuropsychiatric drugs in the past one month; diagnoses of heart diseases, lung diseases, kidney diseases, hypertension, or diabetes; and illiteracy were also grounds for exclusion.

### 2.2. Groups

All subjects were divided into four groups: healthy controls (*n* = 40, group 1), cirrhosis without HE (*n* = 52, group 2), cirrhosis with MHE (*n* = 21, group 3), and cirrhosis with OHE (*n* = 19, group 4). After completion of all the examinations on the day of selection, patients in group 3 were randomized into one of the two subgroups, the control group (*n* = 10, group A) which were given standard liver treatments including hepatoprotective drugs, intravenous infusion of albumin, branched chain amino acids, maintaining fluid, electrolyte and acid-base balance, and prevention of complications for two months, and the treated group (*n* = 11, group B) which were treated by lactulose oral solution (Duphalac, Solvay Pharmaceuticals B.V., Weesp, Netherlands) three times a day, 15 mL at a time, for two months in combination with standard liver treatments. This study was a retrospective, single-blind, and blank randomized controlled trial.

### 2.3. Study Design

Blood analysis was performed on all patients with cirrhosis and included assessment of ammonia, creatinine (Cr), total bilirubin (TBil), albumin (ALB), and prothrombin time (PT). Prior to blood collection patients were fasted for 8 hours or more, before 8 mL blood was collected. Serum specimens were used for liver and renal function tests. Anticoagulant plasma by heparin was used for ammonia tests using the VITROS-250 dry chemistry analyzer (Johnson & Johnson, New Brunswick Zwick, New Jersey, USA) and serum Cr, TBil, and ALB tested using the automatic biochemical analyzer (Beckman, Fullerton, California, USA). The levels of ammonia were retested in group A and group B following two months of treatment. Using a combination of ascites and stage of HE, we determined the grades and scores of Child-Pugh.

In order to confirm diagnosis of MHE, subjects without OHE were administered Number Connection Test-A (NCT-A) and Digit Symbol Test (DST) in a quiet environment prior to TCD examinations. Abnormal results in both tests indicated MHE diagnosis, where abnormity of NCT-A is the mean plus twice the standard deviation, while DST is the mean minus twice the standard deviation [1].

Following biochemical examinations and NCT-A and DST tests, all subjects were subjected to TCD examinations. We used TC8080 TCD diagnostic instrument (Elektromaschinenbau Ettlingen Maschinenfabrik Clasen GmbH, Ettlingen, Baden-Württemberg, Germany) to detect the right MCA by placing a 2 MHz pulsed Doppler probe on the temporal temple. An experienced clinician performed this examination. Peak systolic velocity (*V*_*p*_), mean velocity (*V*_*m*_), end diastolic velocity (*V*_*d*_) as well as the pulsatility index (PI), which reflects cerebrovascular compliance and elasticity, and resistance index (RI), which reflects cerebrovascular diastolic, systolic, and resistant status of the right MCA, of each subject were measured. Finally, after two months of treatment, we again assessed the improvements of these indexes in group A and group B patients.

### 2.4. Statistical Analysis

Data derived from descriptive statistical analysis are presented as percentages for categorical variables and mean ± SD for continuous data and all data were analyzed by SPSS20.0. Measurement data were tested by the Kolmogorov-Smirnov test of normality and Levene test of homogeneity of variance. If the data conformed to normality and homogeneity of variance, they were compared using *t*-test or ANOVA, as appropriate. Pairwise comparisons between multiple groups were performed using SNK-q or LSD-t method of ANOVA. Alternatively, we used Mann-Whitney rank sum test (two groups) or Kruskal-Wallis test (multiple groups). Categorical data were compared by chi-square test. Correlation was analyzed by partial correlation analysis and Pearson's correlation coefficient used for bivariate normal distribution data or Spearman's rank correlation coefficient. A *P* value < 0.05 was considered statistically significant.

## 3. Results

### 3.1. Comparison of Patient Demographics and Blood Analysis before Treatment

**Figure 1:**
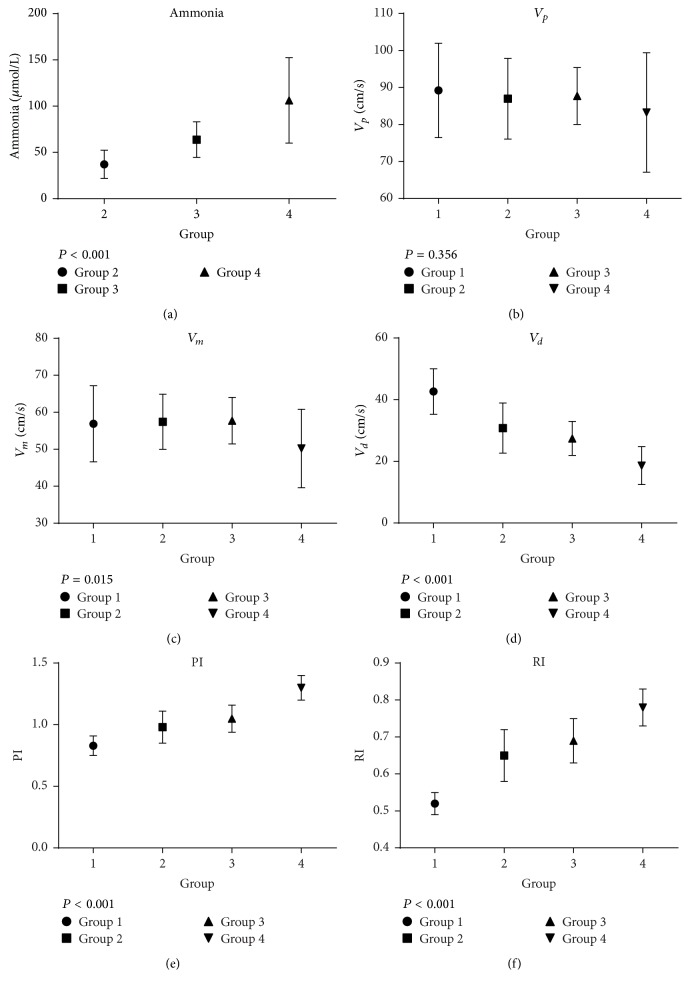
*Comparison of ammonia and TCD among groups 1, 2, 3, and 4 before treatment*. It includes the subfigures (a, b, c, d, e, and f). Subfigure (a) is comparison of ammonia among groups 2, 3, and 4. Subfigure (b) is comparison of *V*_*p*_ among groups 1, 2, 3, and 4. Subfigure (c) is comparison of *V*_*m*_ among groups 1, 2, 3, and 4. Subfigure (d) is comparison of *V*_*d*_ among groups 1, 2, 3, and 4. Subfigure (e) is comparison of PI among groups 1, 2, 3, and 4. Subfigure (f) is comparison of RI among groups 1, 2, 3, and 4. Group 1 is healthy controls group, group 2 is cirrhosis without HE group, group 3 is cirrhosis with MHE group, and group 4 is cirrhosis with OHE group. The data were compared using ANOVA and presented as mean ± SD. *P* < 0.05 means significant differences in an index among groups.

In cirrhotic patients, the incidence of MHE was 22.8% compared to 20.7% in patients with OHE. The prevalence of MHE in Child-Pugh A was 19.0%, 38.1% in Child-Pugh B and the highest in Child-Pugh C at 42.9%. No statistically significant differences in age, sex, educational years, etiology of cirrhosis, Child-Pugh grading, Cr, TBil, ALB, and PT (*P* > 0.05) were observed, while there were significant differences in Child-Pugh scores (0.009) and ammonia (*P* < 0.001) in all groups before treatment (Table 2). Interestingly, ammonia levels appeared to parallel the severity of HE, as ammonia was lowest in cirrhosis patients without HE (37.12 ± 15.28), greater in MHE patients (63.81 ± 19.22), and highest in the OHE group (106.21 ± 46.14) (Figure 1).

Before treatment, there were no statistically significant differences in age, sex, educational years, etiology, Child-Pugh grading, Child-Pugh scores, ammonia, Cr, TBil, ALB, PT, NCT-A, and DST between group A and group B (Table 3).

### 3.2. Comparison of TCD before Treatment

Prior to treatment, no significant differences were observed among group 1, group 2, group 3, and group 4 in *V*_*p*_ (*P* = 0.356) of right MCA, while profound differences were observed in *V*_*m*_ (*P* = 0.015), *V*_*d*_ (*P* < 0.001), PI (*P* < 0.001), and RI (*P* < 0.001) among these four groups (Table 4) (Figure 1). Pairwise comparison of *V*_*m*_ within groups 1 to 3 did not reveal any significant differences; however, we observed important differences with group 4 (*P*_1,2_ = 0.776, *P*_1,3_ = 0.730, *P*_1,4_ = 0.007, *P*_2,3_ = 0.898, *P*_2,4_ = 0.003, and *P*_3,4_ = 0.008). Further pairwise comparison of *V*_*d*_ within all groups demonstrated statistical significance, with no significant differences observed between groups 2 and 3 (*P*_1,2_ < 0.001, *P*_1,3_ < 0.001, *P*_1,4_ < 0.001, *P*_2,3_ = 0.075, *P*_2,4_ < 0.001, and *P*_3,4_ < 0.001). Additionally, PI paralleled the severity of HE as PI was 0.83 ± 0.08 in group 1, 0.98 ± 0.13 in group 2, 1.05 ± 0.11 in group 3, and 1.30 ± 0.10 in group 4 (*P*_1,2_ < 0.001, *P*_1,3_ < 0.001, *P*_1,4_ < 0.001, *P*_2,3_ = 0.024, *P*_2,4_ < 0.001, and *P*_3,4_ < 0.001). Furthermore, the same was observed of RI (0.52 ± 0.03, 0.65 ± 0.07, 0.69 ± 0.06, 0.78 ± 0.05, *P*_1,2_ < 0.001, *P*_1,3_ < 0.001, *P*_1,4_ < 0.001, *P*_2,3_ = 0.011, *P*_2,4_ < 0.001, and *P*_3,4_ < 0.001) (Table 5).

### 3.3. Comparison between Control and Treatment Groups before and after Treatment

**Figure 2:**
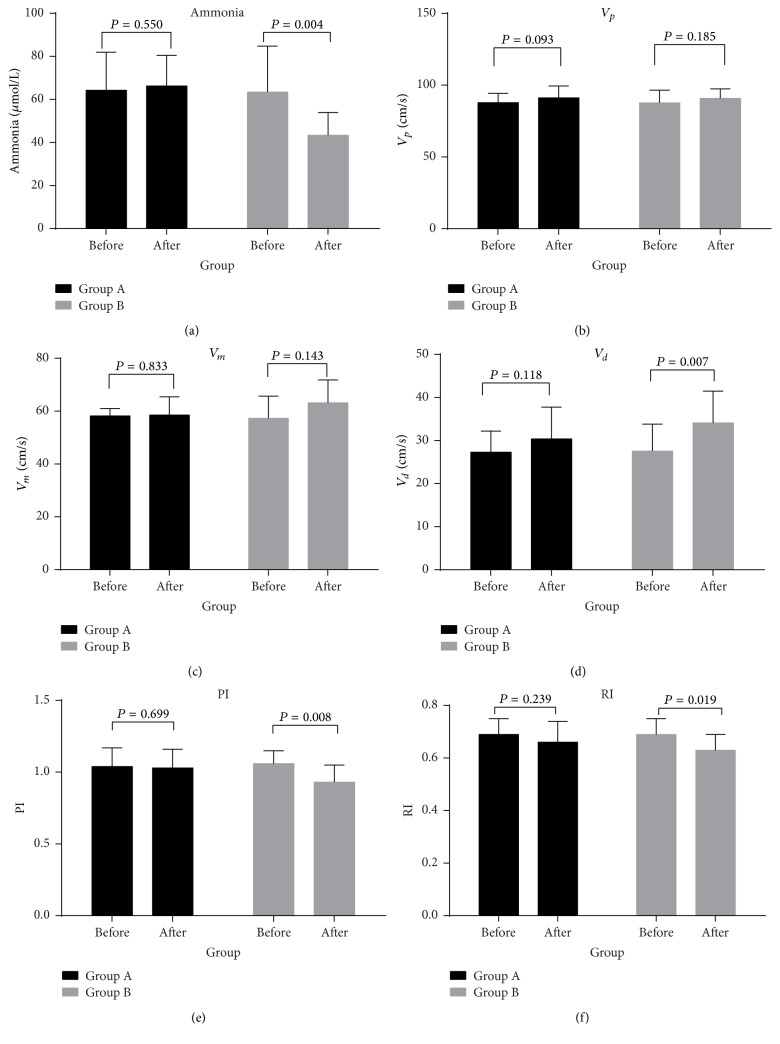
*Comparison of ammonia and TCD before and after treatment in groups A and B*. It includes the subfigures (a, b, c, d, e, and f). Subfigure (a) is comparison of ammonia. Subfigure (b) is comparison of *V*_*p*_. Subfigure (c) is comparison of *V*_*m*_. Subfigure (d) is comparison of *V*_*d*_. Subfigure (e) is comparison of PI. Subfigure (f) is comparison of RI. Group A is the control group; group B is the treated group. The data were compared using *t*-test. *P* < 0.05 means significant differences in an index among groups.

In controls (group A), comparison of ammonia and TCD revealed no significant differences before or after treatment (*P* > 0.05) (Table 6, Figure 2). However, in the treatment group (group B) patients demonstrated a decrease in ammonia following treatment (from 63.45 ± 21.33 to 43.36 ± 10.63, *P* = 0.004). In addition, we observed that *V*_*d*_ increased (from 27.55 ± 6.27 to 34.09 ± 7.38, *P* = 0.007), whilst PI (from 1.06 ± 0.09 to 0.93 ± 0.12, *P* = 0.008) and RI (from 0.69 ± 0.06 to 0.63 ± 0.06, *P* = 0.019) declined. No statistically significant differences were observed in *V*_*p*_ or *V*_*m*_ (*P* = 0.185, *P* = 0.143) (Table 6, Figure 2).

### 3.4. Correlation Analysis among Ammonia, Cerebral Hemodynamics, and Declining Cognitive Function in Patients with MHE

**Figure 3:**
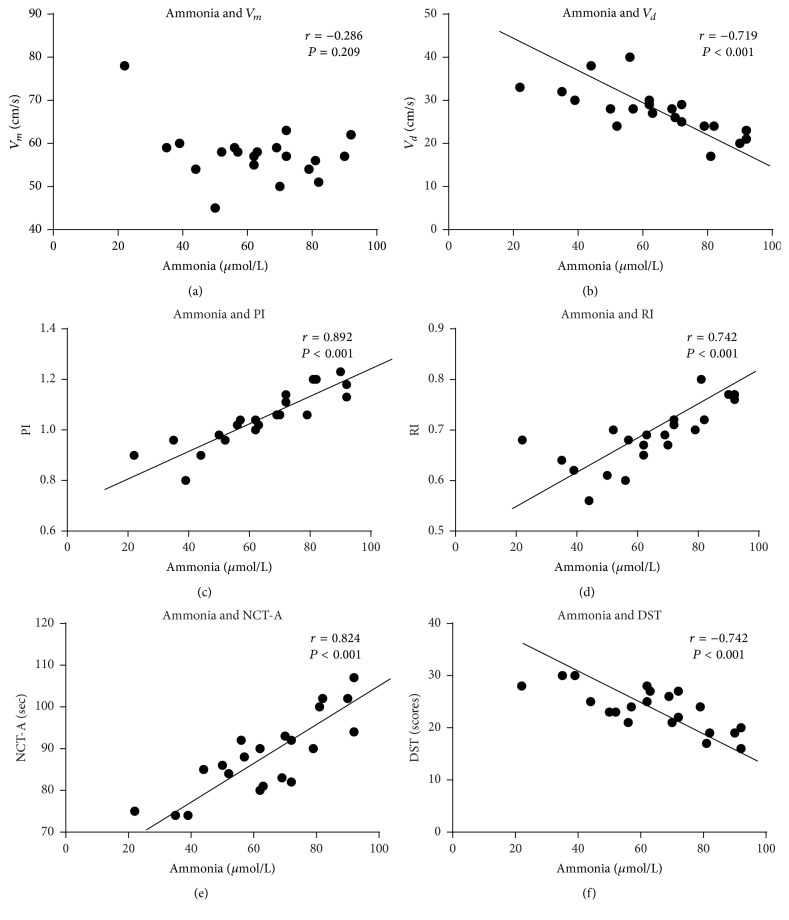
*Correlation between ammonia and cerebral hemodynamics (V*_*m*_, *V*_*d*_*, PI, *and* RI) *and* declining cognitive function (NCT-A and DST) in patients with MHE*. It includes the subfigures (a, b, c, d, e, and f). Subfigure (a) is correlation between ammonia and *V*_*m*_. Subfigure (b) is correlation between ammonia and *V*_*d*_. Subfigure (c) is correlation between ammonia and PI. Subfigure (d) is correlation between ammonia and RI. Subfigure (e) is correlation between ammonia and NCT-A. Subfigure (f) is correlation between ammonia and DST. Correlation was analyzed by Pearson's correlation coefficient. *P* < 0.05 means significant differences in correlation between two indexes.

In patients with MHE, there was no correlation observed between ammonia and *V*_*m*_ (*r* = −0.286, *P* = 0.209), but there was positive correlation between ammonia and PI (*r* = 0.892, *P* < 0.001), RI (*r* = 0.742, *P* < 0.001), and also NCT-A (*r* = 0.824, *P* < 0.001). In contrast, there was negative correlation between ammonia and *V*_*d*_ (*r* = −0.719, *P* < 0.001), DST (*r* = −0.742, *P* < 0.001) (Figure 3).

**Figure 4:**
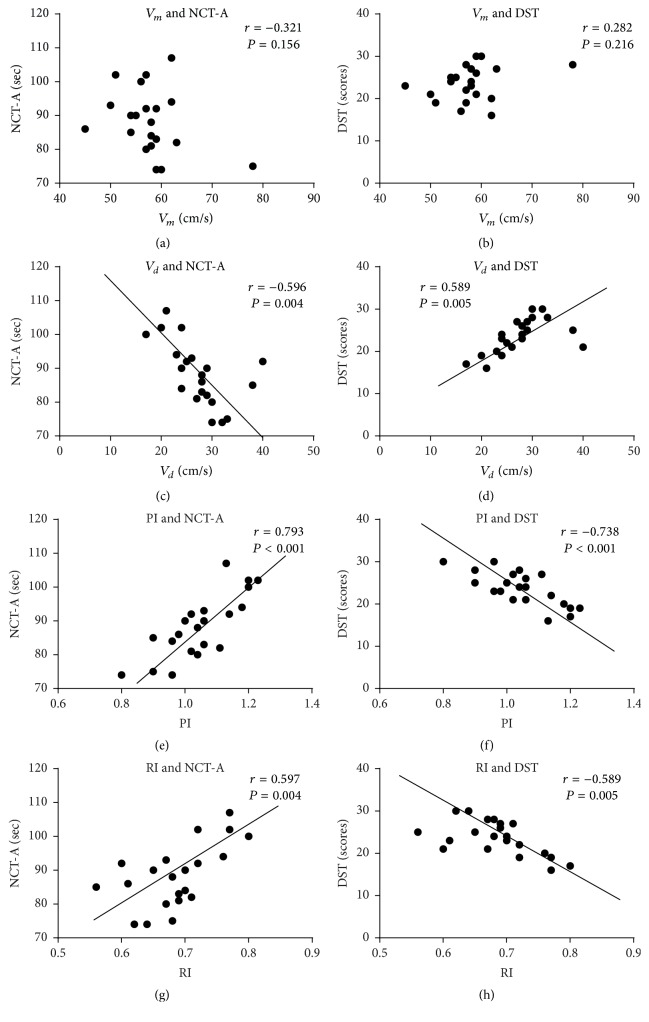
*Correlation between cerebral hemodynamics (V*_*m*_, *V*_*d*_*, PI, *and* RI) and declining cognitive function (NCT-A and DST) in patients with MHE*. It includes the subfigures (a, b, c, d, e, f, g, and h). Subfigure (a) is correlation between *V*_*m*_ and NCT-A. Subfigure (b) is correlation between *V*_*m*_ and DST. Subfigure (c) is correlation between *V*_*d*_ and NCT-A. Subfigure (d) is correlation between *V*_*d*_ and DST. Subfigure (e) is correlation between PI and NCT-A. Subfigure (f) is correlation between PI and DST. Subfigure (g) is correlation between RI and NCT-A. Subfigure (h) is correlation between RI and DST. Correlation was analyzed by Pearson's correlation coefficient. *P* < 0.05 means significant differences in correlation between two indexes.

In patients with MHE, there was no correlation observed between *V*_*m*_ and NCT-A (*r* = −0.321, *P* = 0.156), DST (*r* = 0.282, *P* = 0.216); however we did detect a correlation between *V*_*d*_ and NCT-A (*r* = −0.596, *P* = 0.004), DST (*r* = 0.589, *P* = 0.005), which was opposite to PI (*r* = 0.793, *P* < 0.001; *r* = −0.738, *P* < 0.001) and RI (*r* = 0.597, *P* = 0.004; *r* = −0.589, *P* = 0.005) (Figure 4).

## 4. Discussion

To date, the pathogenesis of HE has not been fully elucidated. It has been proposed that HE is the result of multiple factors and it is widely believed that it is the collective effects by severe dysfunction of organism metabolism and toxic accumulation, amongst which elevated ammonia has emerged as one of the main pathogeneses [10, 11].

In severe liver diseases, liver dysfunction, reduced transformation, and clearance of metabolites by liver, coupled with overgenerating of ammonia, cause significant increasing of blood ammonia and it cannot be excluded from the body. Ammonia has toxic effects on the nervous system. (1) Ammonia enters the CNS through the blood-brain barrier (BBB) and astrocytes are the only cells in the brain that can metabolize ammonia [12] which play an important role in maintaining the balance of BBB, autoregulation function of CNS, and cerebral blood flow [13]. Synthetizing of glutamine by ammonia and glutamic acid results in obvious increasing of glutamine in astrocytes, causing conduction disorders of glutamatergic neurons, astrocytes swelling, cerebral edema, and intracranial hypertension [14, 15]. Long-time exposure of astrocytes in high concentration of ammonia will lead to compensatory loss of permeating substances such as taurine and inositol, so that the astrocytes become very sensitive to the edema effect caused by other HE-induced factors and then induce HE or result in sudden deterioration of HE [16–18]. (2) Ammonia can bring about energy metabolism obstacles of carbohydrate utilization, glycolysis, tricarboxylic acid cycle, and ATP synthesis and utilization and result in insufficiency of brain energy supply; thus it cannot maintain the excitement of the CNS [19]. (3) Ammonia may decrease the activity of Na^+^-K^+^-ATPase in CNS, weakening the function of BBB enzyme system, leading to the unblocking of toxic substances into CNS through the damaged BBB and impairing the autoregulation function of intracranial blood flow. Ammonia may also compete with K^+^ for binding to Na^+^-K^+^-ATPase in neuronal cells and astrocytes membrane, leading to conduction disturbances of nerve impulse and astrocytes swelling or brain edema [20]. (4) Ammonia can act directly on the nerve membrane, causing postsynaptic inhibition and interference with nerve action potential. Glutamine receptors activity on the postsynaptic plate will reduce and glutamine carriers on the astrocytic membrane will inactivate, thereby leading to astrocytes swelling [21]. Over time, these cells transform into Alzheimer type II astrocytes [22, 23]. (5) In addition, ammonia can lead to imbalance of inhibitory and excitatory neurotransmitter, increasing the contents of inhibitory neurotransmitter; it will change gene expression of astrocytes structural protein, glial fibrillary acidic protein, aquaporin 4, and so forth, so that proteins maintain the normal function of the brain alter abnormally [24]; it may also increase calcium influx in astrocytes and start oxidation and nitrification stress directly, resulting in mitochondrial dysfunction and energy loss, causing inflammation and damaging intracellular signaling pathways, and ultimately leading to nervous system dysfunction [25–27].

Although our study found that there were significant differences in ammonia (*P* < 0.001) amongst all experimental groups and that ammonia paralleled the severity of HE, we demonstrate that ammonia levels had significant overlap in different grades of HE consistent with the observations in Blei and Córdoba's study [28]. This overlap may be related to the generation, absorption, and removal of ammonia which can be affected by a variety of intrahepatic and extrahepatic factors and ammonia may not be the only pathogenesis of HE [29]. Also, clinical testing and experimental studies have shown that patients with hyperammonemia do not necessarily have HE, and not all patients with HE show significantly elevated blood ammonia which requires us to distinguish HE from other diseases [30]. Therefore, the theory of ammonia toxicity alone cannot well explain the pathogenesis of HE, and it should be along with other hypotheses such as inflammatory response, oxidation, and nitrification stress, amino acid metabolism imbalance, neurotransmitter imbalance, and manganese toxicity. Consequently, the clinical value of assessing blood ammonia concentration in HE classification and MHE diagnosis remains unclear. Lactulose is widely used as a clinical treatment for MHE, as it promotes the metabolism of ammonia, by reducing the intestinal absorption leading to an overall reduction in circulating ammonia [31]. In the present study, we treated MHE patients with lactulose for 2 months, resulting in a decline in ammonia levels (from 63.45 ± 21.33 to 43.36 ± 10.63, *P* = 0.004), indicating that lactulose is an effective treatment for MHE via the compounds ability to reduce ammonia.

Current literatures demonstrated a good understanding of cerebral hemodynamics, cerebral metabolism, and cerebral autoregulation function in acute/fulminant hepatic failure, whilst their role in HE and chronic liver disease/cirrhosis remains poorly understood. Felipo et al. found that determining noninvasive brain blood flow using arterial spin labeling (ASL) could detect MHE earlier than Psychometric Hepatic Encephalopathy Score (PHES) [32] indicating there are early changes to cerebral hemodynamics in the preliminary stages of HE. In the present study, prior to treatment, we observed no differences in *V*_*p*_ (*P* = 0.356) of right MCA, while significant differences were evident in *V*_*m*_, *V*_*d*_, PI, and RI (*P* < 0.05). Furthermore, our results demonstrate that more serious HE was associated with decreased cerebral blood flow velocity, particularly mean velocity and end diastolic velocity. Simultaneously, PI and RI paralleled the severity of HE. As PI reflects the compliance and elasticity of the cerebral vasculature and RI reflects diastolic, systolic, and resistant status of the cerebral vasculature this result suggests that systolic and diastolic cerebral autoregulation is impaired, as PI and RI increased in cirrhotic patients with HE and cerebral hemodynamics was related to severity of HE, consistent with results from previous studies [33, 34].

Many studies have established that cerebral blood flow, cerebral autoregulation, and cerebral edema are improved in acute/fulminant hepatic failure through a series of active treatments [35–37]. However, to date little is known about the changes of cerebral hemodynamics in cirrhotic patients with MHE after treatment. Lactulose can reduce the impact of ammonia on cerebral blood flow and brain function by reducing ammonia and as confirmed in our study may therefore improve cerebral hemodynamics in patients with HE. Following two months of treatment with lactulose, *V*_*d*_ increased (from 27.55 ± 6.27 to 34.09 ± 7.38, *P* = 0.007), whilst PI (from 1.06 ± 0.09 to 0.93 ± 0.12, *P* = 0.008) and RI (from 0.69 ± 0.06 to 0.63 ± 0.06, *P* = 0.019) declined. In addition, the treatment did not significantly alter *V*_*p*_ and *V*_*m*_ (*P* = 0.185, *P* = 0.143). As such, we demonstrate that lactulose treatment not only does improve cerebral blood flow and cerebral autoregulation in acute/fulminant hepatic failure, but also has potential for use in patients with MHE. Furthermore, it was an effective method for disease surveillance, efficacy evaluation by detection, and monitoring of TCD.

Cognition is the mental action and process of acquiring and understanding of knowledge, with the basis of the normal functioning of cerebral cortex. Consequently, any factors including ammonia which cause abnormity in brain function and structure may lead to declining cognitive function. Our study found that, in patients with MHE, there was positive correlation between ammonia and PI, RI, and also NCT-A (*P* < 0.001). In contrast, there was negative correlation between ammonia and *V*_*d*_ and DST (*P* < 0.001). Similarly, there was negative correlation between *V*_*d*_ and NCT-A and positive correlation between *V*_*d*_ and DST (*P* < 0.05), which was opposite to PI and RI (*P* < 0.05). This indicated that there were tight correlations among ammonia, reduced cerebral blood flow velocity, impaired systolic and diastolic cerebral autoregulation, and declining cognitive function in patients with MHE.

However, TCD application is still associated with some issues such as the impact of different operators and lack of uniform criteria about normal and abnormal values. The failure rate of TCD is 2.7% to 5% in older patients, who often have thickening of the skull, arterial tortuosity, and arterial shift [7]. The present study addressed changes in cerebral hemodynamics in patients with HE as well as its role in monitoring severity of HE and efficacy assessment. Future studies will need to further explore the critical value for diagnosis and classification. Although this study reports some important findings in the improvement of cerebral hemodynamics after 2 months of treatment, further long-term follow-up of such patients is required to better understand these findings.

The incidence of MHE in cirrhotic patients has been reported to be from 30% to 84% according to foreign data [38–40] while it is between 29.2% and 57.1% in China [1] and 22.8% in the present study. These differences may be due to the lack of uniform diagnostic criteria of MHE, the different operators, sample size, and individual and regional differences. It has been reported by Groeneweg et al. that cirrhotic patients of Child-Pugh A had a low prevalence (15%) of MHE, while MHE was present in half of the patients with advanced cirrhosis (B/C) [41]. Our study confirmed there was a significant difference in the prevalence of MHE amongst these groups, with the lowest incidence observed in Child-Pugh A (19%); MHE had a higher incidence in Child-Pugh B (38.1%) and C (42.9%) groups. Accordingly, patients' liver function should be taken into account in the diagnosis of MHE and more attention directed to cirrhotic patients without OHE clinically, especially in patients with Child-Pugh B and Child-Pugh C.

Patients with MHE do not demonstrate any overt clinical symptoms of HE but do exhibit mild cognitive and psychomotor deficits [42]. It has been previously reported that the majority of patients with MHE have changes in memory, reaction, attention, and behavior, which may impact their work and personal life to different degrees. Approximately 60% of patients with MHE have been found to be unfit for work and approximately 50% unfit to drive [43]. However, due to the subtle nature of MHE symptoms, preventative and treatment strategies are difficult to implement. Therefore, a better understanding of MHE pathology may lead to improvements in early diagnosis, treatment, and disease surveillance to significantly improve the quality of life of MHE patients.

## 5. Conclusion

Our study reports that, in patients with HE, ammonia, PI, and RI increased and cerebral blood flow velocity slowed down. At the same time, ammonia, PI, and RI paralleled the severity of HE, which indicated reduced cerebral blood flow and impairment of systolic and diastolic cerebral autoregulation in patients with HE. There were tight correlations among ammonia, reduced cerebral blood flow velocity, impaired systolic and diastolic cerebral autoregulation, and declining cognitive function in patients with MHE. After treatment with lactulose, ammonia level, PI, and RI decreased significantly while *V*_*d*_ increased. A better understanding of these parameters in the context of HE may provide an important basis for disease surveillance and the development of new treatment strategies to improve cerebral hemodynamics. The results of this study highlight the importance of correct diagnosis, monitoring, and treatment of patients with HE, particularly MHE.

## Figures and Tables

**Table 1:** Cirrhotic patient demographics.

Variables	Mean/proportion
Age (year)	58.91 ± 8.59
Sex (M/F)	54/38
Educational years	9.74 ± 3.17
Etiology	
B viral hepatitis cirrhosis	40 (43.5%)
C viral hepatitis cirrhosis	3 (3.2%)
Primary biliary cirrhosis	7 (7.6%)
Autoimmune hepatitis cirrhosis	8 (8.7%)
Schistosomiasis cirrhosis	8 (8.7%)
Cryptogenic cirrhosis	26 (28.3%)
Child-Pugh grading	
Grade A	24 (26.1%)
Grade B	36 (39.1%)
Grade C	32 (34.8%)
Child-Pugh scores	8.87 ± 2.40

Table 1 presents cirrhotic patient demographics including age, sex, educational years, etiology, Child-Pugh grading, and Child-Pugh scores before treatment. Data derived from descriptive statistical analysis were presented as percentages for categorical variables and mean ± SD for continuous data.

**Table 2:** Patient demographics and blood analysis before treatment among groups 1, 2, 3, and 4.

Variables	Group 1	Group 2	Group 3	Group 4	*F*/*χ*^2^ value	*P*
Cases	40	52	21	19	—	—
Age (year)	54.32 ± 10.14	58.31 ± 9.24	59.10 ± 7.55	60.37 ± 8.05	2.587	0.056
Sex (M/F)	24/16	31/21	12/9	11/8	0.064	0.996
Educational years	9.55 ± 3.37	10.37 ± 3.33	9.19 ± 2.87	8.63 ± 2.75	1.662	0.178
Etiology					7.148	0.711
B viral hepatitis cirrhosis	—	21	11	8		
C viral hepatitis cirrhosis	—	1	1	1		
Primary biliary cirrhosis	—	3	3	1		
Autoimmune hepatitis cirrhosis	—	5	0	3		
Schistosomiasis cirrhosis	—	6	1	1		
Cryptogenic cirrhosis	—	16	5	5		
Child-Pugh grading					7.229	0.124
Grade A	—	18	4	2		
Grade B	—	21	8	7		
Grade C	—	13	9	10		
Child-Pugh scores	—	8.27 ± 2.36	9.19 ± 2.16	10.16 ± 2.27	4.965	0.009
Ammonia (*μ*mol/L)	—	37.12 ± 15.28	63.81 ± 19.22	106.21 ± 46.14	52.154	<0.001
Cr (*μ*mol/L)	—	86.01 ± 22.80	83.35 ± 40.11	79.14 ± 43.92	0.318	0.728
TBil (*μ*mol/L)	—	45.78 ± 16.76	45.57 ± 14.51	42.81 ± 14.83	0.254	0.776
ALB (g/L)	—	29.62 ± 7.92	28.38 ± 5.36	31.05 ± 6.71	0.691	0.504
PT (sec)	—	3.14 ± 1.76	2.87 ± 1.74	3.25 ± 1.76	0.263	0.769
NCT-A (sec)	46.92 ± 11.80	51.31 ± 10.93	88.29 ± 9.38	—	108.840	<0.001
DST (scores)	52.35 ± 10.69	49.98 ± 9.98	23.57 ± 4.04	—	72.476	<0.001

Table 2 indicates comparison of age, sex, educational years, etiology, Child-Pugh grading, Child-Pugh scores, ammonia, Cr, TBil, ALB, PT, NCT-A, and DST among groups 1, 2, 3, and 4 before treatment. Group 1 is healthy controls group, group 2 is cirrhosis without HE group, group 3 is cirrhosis with MHE group, and group 4 is cirrhosis with OHE group. Continuous data were compared using ANOVA and categorical data were compared by a chi-square test. *P* < 0.05 means significant differences in an index among groups.

**Table 3:** Patient demographics and blood analysis before treatment between groups A and B.

Variables	Group A	Group B	*t*/*χ*^2^ value	*P*
Cases	10	11	—	—
Age (year)	58.10 ± 6.67	60.00 ± 8.49	−0.566	0.578
Sex (M/F)	6/4	6/5	0.064	0.801
Educational years	9.00 ± 2.83	9.36 ± 3.04	−0.283	0.780
Etiology			2.582	0.630
B viral hepatitis cirrhosis	5	6		
C viral hepatitis cirrhosis	1	0		
Primary biliary cirrhosis	2	1		
Autoimmune hepatitis cirrhosis	0	0		
Schistosomiasis cirrhosis	0	1		
Cryptogenic cirrhosis	2	3		
Child-Pugh grading			0.565	0.754
Grade A	2	2		
Grade B	5	4		
Grade C	3	5		
Child-Pugh scores	9.10 ± 2.42	9.27 ± 2.01	−0.179	0.860
Ammonia (*μ*mol/L)	64.30 ± 17.07	63.45 ± 21.33	0.098	0.923
Cr (*μ*mol/L)	89.00 ± 45.63	78.22 ± 35.82	0.605	0.552
TBil (*μ*mol/L)	42.53 ± 14.14	48.34 ± 14.94	−0.912	0.373
ALB (g/L)	29.40 ± 7.03	27.45 ± 3.33	0.824	0.420
PT (sec)	2.98 ± 2.17	2.76 ± 1.34	0.278	0.784
NCT-A (sec)	87.20 ± 7.83	89.27 ± 10.89	−0.496	0.626
DST (scores)	24.20 ± 3.36	23.00 ± 4.67	0.670	0.511

Table 3 indicates comparison of age, sex, educational years, etiology, Child-Pugh grading, Child-Pugh scores, ammonia, Cr, TBil, ALB, PT, NCT-A, and DST between groups A and B before treatment. Group A is the control group; group B is the treated group. Continuous data were compared using *t*-test and categorical data were compared by a chi-square test. *P* < 0.05 means significant differences in an index between two groups.

**Table 4:** Comparison of TCD before treatment.

Variables	Group 1	Group 2	Group 3	Group 4	*F* value	*P*
*V*_*p*_ (cm/s)	89.22 ± 12.76	86.98 ± 10.90	87.71 ± 7.67	83.26 ± 16.14	1.089	0.356
*V*_*m*_ (cm/s)	56.90 ± 10.29	57.42 ± 7.46	57.71 ± 6.29	50.21 ± 10.59	3.608	0.015
*V*_*d*_ (cm/s)	42.65 ± 7.37	30.80 ± 8.13	27.43 ± 5.52	18.63 ± 6.15	53.131	<0.001
PI	0.83 ± 0.08	0.98 ± 0.13	1.05 ± 0.11	1.30 ± 0.10	81.599	<0.001
RI	0.52 ± 0.03	0.65 ± 0.07	0.69 ± 0.06	0.78 ± 0.05	98.676	<0.001

Table 4 describes comparison of TCD including *V*_*p*_, *V*_*m*_, *V*_*d*_, PI, and RI among groups 1, 2, 3, and 4 before treatment. Group 1 is healthy controls group, group 2 is cirrhosis without HE group, group 3 is cirrhosis with MHE group, and group 4 is cirrhosis with OHE group. Continuous data were compared using ANOVA. *P* < 0.05 means significant differences in an index among groups.

**Table 5:** Pairwise comparison of TCD before treatment.

Variables	*P*_1,2_	*P*_1,3_	*P*_1,4_	*P*_2,3_	*P*_2,4_	*P*_3,4_
*V*_*p*_ (cm/s)	0.373	0.640	0.076	0.813	0.248	0.242
*V*_*m*_ (cm/s)	0.776	0.730	0.007	0.898	0.003	0.008
*V*_*d*_ (cm/s)	<0.001	<0.001	<0.001	0.075	<0.001	<0.001
PI	<0.001	<0.001	<0.001	0.024	<0.001	<0.001
RI	<0.001	<0.001	<0.001	0.011	<0.001	<0.001

Table 5 presents pairwise comparison of TCD including *V*_*p*_, *V*_*m*_, *V*_*d*_, PI, and RI among groups 1, 2, 3, and 4 before treatment. *P*_1,2_ indicates comparison between group 1 and group 2, *P*_1,3_ means comparison between group 1 and group 3, *P*_1,4_ compares group 1 and group 4, *P*_2,3_ refers to a comparison between group 2 and group 3, *P*_2,4_ indicates a comparison between group 2 and group 4, and *P*_3,4_ means comparison between group 3 and group 4. Group 1 is healthy controls group, group 2 is cirrhosis without HE group, group 3 is cirrhosis with MHE group, and group 4 is cirrhosis with OHE group. Pairwise comparisons between multiple groups were performed using SNK-q or LSD-t method of ANOVA. *P* < 0.05 means significant differences in an index between two groups.

**Table 6:** Comparison of ammonia and TCD before and after treatment.

Variables	Group A		Group B		*t*_1_ value	*t*_2_ value	*P*_1_	*P*_2_
	Before	After	Before	After				
Ammonia (*μ*mol/L)	64.30 ± 17.67	66.30 ± 14.19	63.45 ± 21.33	43.36 ± 10.63	−0.621	3.760	0.550	0.004
*V*_*p*_ (cm/s)	87.80 ± 6.48	91.20 ± 8.24	87.64 ± 8.94	90.82 ± 6.59	−1.880	−1.423	0.093	0.185
*V*_*m*_ (cm/s)	58.20 ± 2.82	58.60 ± 6.80	57.27 ± 8.46	63.18 ± 8.65	−0.217	−1.590	0.833	0.143
*V*_*d*_ (cm/s)	27.30 ± 4.90	30.40 ± 7.35	27.55 ± 6.27	34.09 ± 7.38	−1.730	−3.404	0.118	0.007
PI	1.04 ± 0.13	1.03 ± 0.13	1.06 ± 0.09	0.93 ± 0.12	0.399	3.333	0.699	0.008
RI	0.69 ± 0.06	0.66 ± 0.08	0.69 ± 0.06	0.63 ± 0.06	1.262	2.785	0.239	0.019

Table 6 indicates comparison of ammonia and TCD including *V*_*p*_, *V*_*m*_, *V*_*d*_, PI, and RI between group A and B before and after treatment. *t*_1_ and *P*_1_ represent comparison before and after treatment in group A; *t*_2_ and *P*_2_ represent comparison before and after treatment in group B. Group A is the control group; group B is the treated group. Continuous data were compared using *t*-test. *P* < 0.05 means significant differences in an index before and after treatment.
